# Osteomyelitis Masquerading as Cellulitis: A Case Report

**DOI:** 10.7759/cureus.53238

**Published:** 2024-01-30

**Authors:** Sanjiya Arora, Vikramaditya Rai, Devendra Tripathi, Sachin M Chaudhary, Ajay Singh, Mohitha Chowdary

**Affiliations:** 1 Internal Medicine, Sub-Divisional Hospital (SDH) cum Civil Hospital, Fatehabad, IND; 2 Orthopedics, Dr. Rajendra Prasad Government Medical College & Hospital, Kangra, IND; 3 Internal Medicine, New York Medical College, Valhalla, USA; 4 Internal Medicine, Gujarat Cancer Society (GCS) Medical College, Hospital and Research Centre, Ahmedabad, IND; 5 Internal Medicine, Shri Ram Murti Smarak Institute of Medical Sciences, Bareilly, IND; 6 Internal Medicine, Sri Venkateswara Institute of Medical Sciences (SVIMS), Tirupati, IND

**Keywords:** osteopenia, antimicrobial therapy, debridement, cellulitis, osteomyelitis

## Abstract

Osteomyelitis, a significant global healthcare issue, often results from infections related to open fractures, surgery, or conditions like diabetic foot ulcers. This report describes a case of osteomyelitis in a 62-year-old female with various pre-existing health conditions. The patient initially presented with swelling, pain, and difficulty walking in the right lower extremity, accompanied by systemic symptoms. Despite an initial diagnosis of cellulitis and treatment with ceftriaxone, a subsequent CT scan revealed a pretibial abscess and confirmed osteomyelitis caused by pan-sensitive *Escherichia coli*. Surgical debridement was performed, and the patient received six weeks of intravenous antibiotics. Hence, a heightened level of suspicion is essential to facilitate a timely diagnosis of osteomyelitis and enhance long-term prognosis. The case underscores the importance of a multidisciplinary approach, including meticulous surgical intervention and tailored antimicrobial therapy, in achieving positive outcomes for osteomyelitis patients.

## Introduction

Osteomyelitis (OM) of the lower limb represents a large global healthcare burden. It often arises from a contiguous focus of infection and is a recognized complication of open fractures or their surgical treatment, arthroplasty, and diabetic foot ulcers [1]. Historically, this debilitating condition is associated with high rates of recurrence and secondary amputation. With an estimated frequency of 21.8 cases per 100,000 person-years in the United States, OM is a rather uncommon illness [2]. However, excellent long-term outcomes are now achieved by adopting a multidisciplinary approach.

Based on the patient's clinical presentation, inflammatory indicators, tissue samples for microbiological analysis, and pertinent imaging, such as MRIs and X-rays, the diagnosis is made. The treatment involves meticulous surgical debridement, skeletal and soft tissue reconstruction, and a tailored antimicrobial regimen. Any bone can get infected; however, due to trauma, reduced vascularity, and ambulatory pressure, the lower limbs are typically more impacted than the upper limbs due to the etiology and pathophysiology of infection [3]. Among the most common pathogens is *Staphylococcus aureus*. Children and vulnerable individuals are the most typical targets.

In this report, we present a challenging case involving an elderly individual whose diagnosis posed difficulty due to the manifestation of OM presenting with clinical features typical of cellulitis.

## Case presentation

A 62-year-old female patient with a medical history of multiple comorbidities presented in our clinic with chronic obstructive pulmonary disease (COPD) on 4L and heart failure with preserved ejection fraction (HFpEF). She had a previous diagnosis of polymyalgia rheumatica for which she was on long-term low-dose steroids. She also had hypersensitivity lung disease (HLD), hypothyroidism, obstructive sleep apnea (OSA), diabetes mellitus (DM) with glycated hemoglobin (HbA1c) 6.7, and depression.

**Figure 1:**
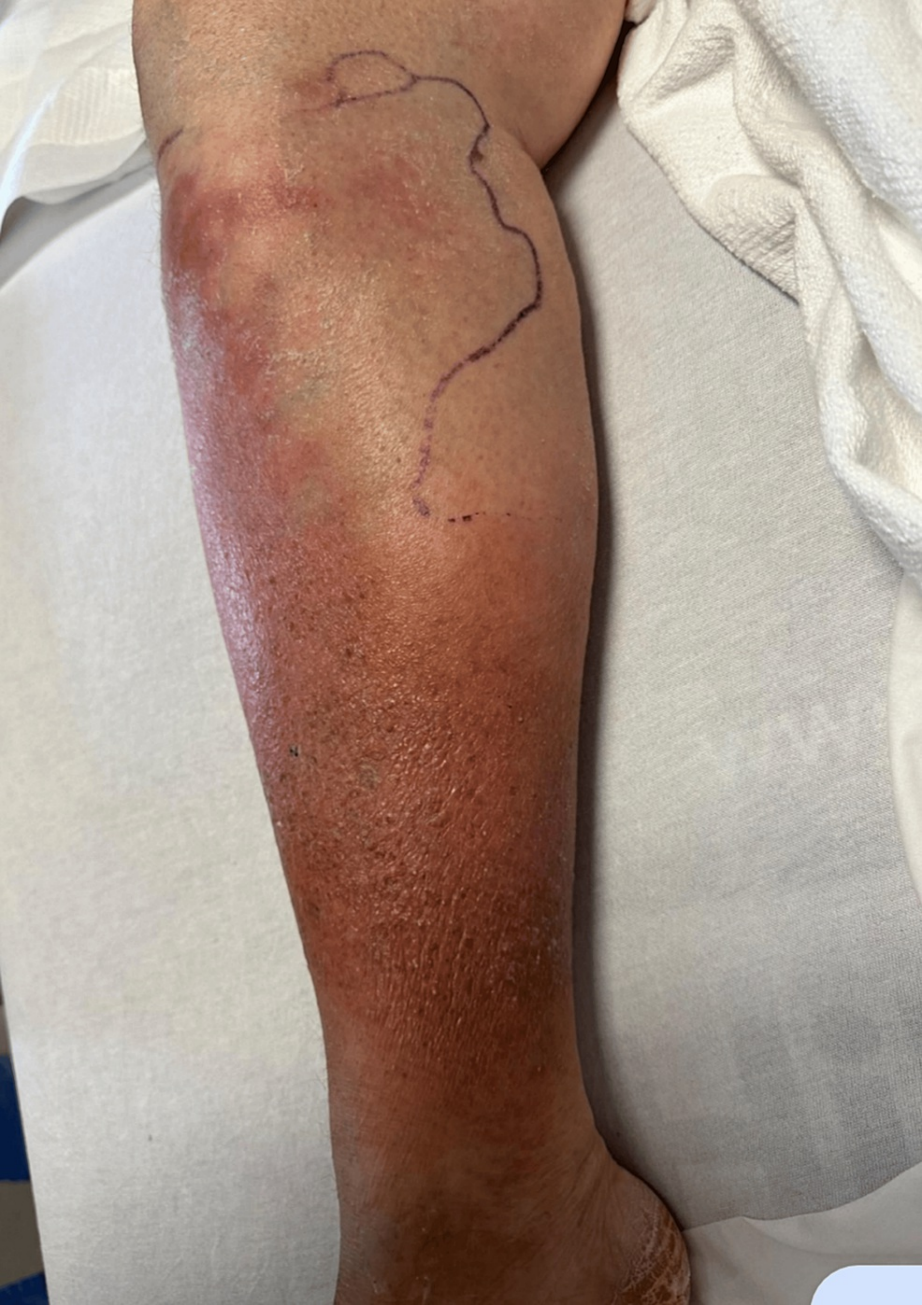
Right lower extremity (RLE) showing erythema, edema, tenderness, and warmth of the proximal anterior shin. The black curved line in the figure is drawn by the physicians for medical reasons.

The patient presented with swelling and pain below the right knee and difficulty in walking accompanied by intermittent episodes of fever, chill, and nausea for the past two weeks. The patient exhibited a cough accompanied by the production of yellow sputum over the preceding three weeks. She had spent three weeks, prior to presentation, in a skilled nursing facility (SNF) after being discharged from another hospital for newly diagnosed HFpEF. Initial physical examination confirmed the patient was afebrile and hemodynamically stable. The right lower extremity (RLE) showed erythema, edema, tenderness, and warmth of the proximal anterior shin (Figure 1). Bilateral lower extremities had chronic venous stasis changes with strong lower extremity pulses laterally. In addition to routine laboratory tests including complete blood count (CBC) and comprehensive metabolic panel (CMP), chest X-ray, CT chest, RLE X-ray, RLE venous and arterial Doppler, electrocardiogram (EKG), erythrocyte sedimentation rate (ESR), and laryngopharyngeal reflux (LRP) were ordered. RLE X-ray showed a normal right tibia and fibula with no suspicious osseous lesion. RLE venous and arterial Doppler showed no evidence of deep vein thrombosis (DVT). There were no acute arterial findings in the RLE or evidence of chronic posterior tibial atherosclerosis.

Chest X-ray showed an asymmetric right lower lobe opacity. Subsequent CT chest showed subsegmental atelectasis in the bilateral lower lobes. EKG showed no evidence of ischemia or arrhythmia. Initial lab tests were pertinent for an elevated ESR of 87, CRP 18.0, procalcitonin 0.39, N-terminal pro-B-type natriuretic peptide (NT-ProBNP) 164, white blood cells (WBC) 11.0, Hb 12.0, platelets 359, thyroid stimulating hormone (TSH) 1.88, glomerular filtration rate (GFR) 108, and normal liver function tests with a mildly decreased albumin of 2.7.

**Figure 2:**
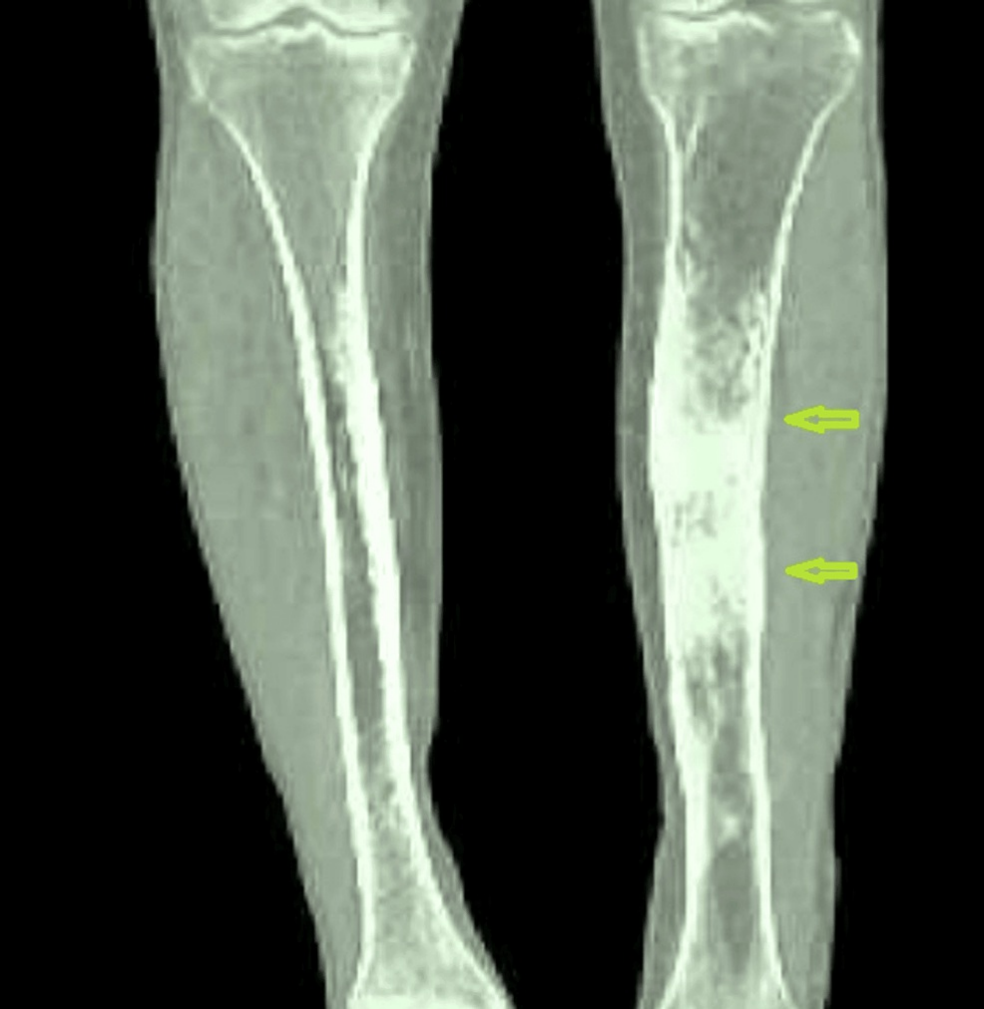
Tibial osteomyelitis CT coronal depicting periostitis and inflammation (infection) in the surrounding tissue.

**Figure 3:**
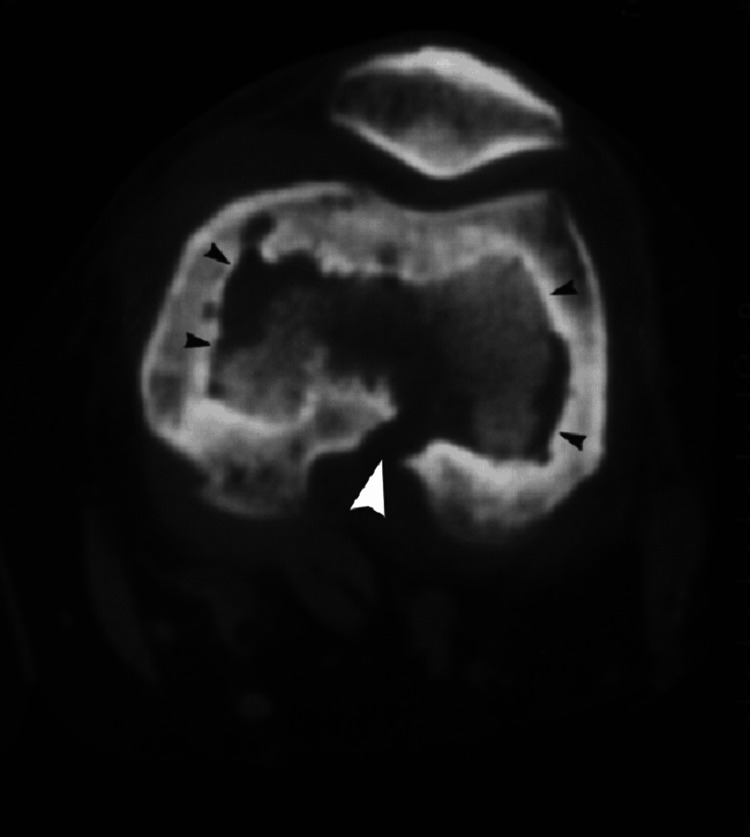
Transaxial CT image shows osteomyelitis of tibia with sequestrum formation.

No periosteal reaction or cortical destruction was observed. She was diagnosed with RLE cellulitis and was put on ceftriaxone. On day 3 of hospitalization, her respiratory symptoms resolved with intravenous (IV) antibiotics and aggressive incentive spirometry, and her RLE cellulitis showed improvement with erythema receding from the original outline. However, on day 4, there was an increase in the patient's RLE pain, swelling, and redness, and the erythema had spread superiorly toward the original outline. Antibiotics were escalated to IV cefazolin and clindamycin. Ultrasound of the medial RLE was obtained and showed no fluid collection or mass. She was maintained on IV cefazolin and oral clindamycin for five more days with a modest improvement of her cellulitis symptoms. On day 15, a lump/hematoma was noticed in the right medial area just inferior to the knee with fluctuant swelling. CT scan was ordered which revealed a pretibial abscess just inferior to the tibial tubercle with cortical involvement of the anterior tibia (Figures 2, 3). There was subcutaneous edema throughout the whole right lower leg. The culture of the abscess confirmed pan-sensitive *Escherichia coli* infection and the laboratory findings were consistent with OM. Orthopedic surgery was consulted for possible debridement. Copious irrigation and tissue debridement were performed. Debridement showed subcutaneous purulence, which was taken for culture, and a full-thickness cortical perforation through the anterior tibia medial to the tibial tubercle. No additional bone resection was needed for adequate debridement due to the perforation. The patient was placed on IV antibiotics for six weeks and intraoperative culture tests showed no growth, gram-negative bacilli, or WBCs.

## Discussion

OM is a bacterial infection-related inflammatory bone disease that primarily affects the lower limbs. Either hematogenous spread or contiguous spread, which includes open trauma and orthopedic procedures, causes infection. The most prevalent pathogen is *S. aureus*. Nevertheless., in our specific case, the primary offender was an *E. coli* infection. The clinical presentation, in conjunction with inflammatory markers, tissue sampling for microbiological examination, and pertinent imaging, such as MRIs and X-rays, are used to make the diagnosis [4]. Based on the acuity and severity of the infection as well as host characteristics (Cierny-Mader classification), the treatment options are conservative, medical (antibiotics), or surgical [5].

Surgical therapy includes radical debridement of the soft tissues and bone that are infected, removal of metalwork if necessary, elimination of dead space, sufficient soft tissue coverage, and sufficient vascular supply to the affected area [6]. For significant bone lesions, reconstruction possibilities include bone transport and "induced membrane" Masquelet methods. Moreover, a longer course of antibiotics is typically advised; most doctors advocate four to six weeks of treatment, depending on the organism, the location, and the effectiveness of debridement [7]. Long-term suppressive antibiotic therapy is a more contentious use of antibiotics, yet it is frequently employed when other options for treatment are exhausted.

Radiographic findings of OM include osteopenia and bone resorption, cortical loss, bony destruction, and periosteal reaction. However, contrary to this, the X-ray findings of the RLE in the present case showed a normal right tibia and fibula with no suspicious osseous lesion and no periosteal reaction or cortical destruction. CT is more sensitive than X-ray for assessing cortical and trabecular integrity, periosteal reaction, intraosseous gas, soft tissue gas, and the extent of sinus tracts and, more importantly, we can see osseous sequestrum and involucrum on CT too [3]. In our case, a CT scan was conducted, revealing a pretibial abscess located just below the tibial tubercle, with cortical engagement of the anterior tibia. However, the preferred diagnostic imaging at less than two weeks would be MRI, which would demonstrate abnormal marrow edema as early as one to five days following the onset of infection [4]. Bone biopsy, percutaneous or open, is recommended.

## Conclusions

It is essential to identify and resolve the predisposing factors that can lead to osteomyelitis. In our case, the reason for the occurrence of chronic osteomyelitis was DM and peripheral neuropathy. Older age, immunosuppression, DM, chronic corticosteroid usage, cancer, malnourishment, untreated prostatitis, intravenous drug use, and, more recently, an increase in neurosurgery procedures are risk factors. Therefore, early diagnosis of OM is essential to prevent complications and surgical interventions.
